# Specific CT 3D rendering of the treatment zone after Irreversible Electroporation (IRE) in a pig liver model: the “Chebyshev Center Concept” to define the maximum treatable tumor size

**DOI:** 10.1186/1471-2342-14-2

**Published:** 2014-01-10

**Authors:** Dominik Vollherbst, Stefan Fritz, Sascha Zelzer, Miguel F Wachter, Maya B Wolf, Ulrike Stampfl, Daniel Gnutzmann, Nadine Bellemann, Anne Schmitz, Jürgen Knapp, Philippe L Pereira, Hans U Kauczor, Jens Werner, Boris A Radeleff, Christof M Sommer

**Affiliations:** 1 Department of Diagnostic and Interventional Radiology, University Hospital Heidelberg, Heidelberg, Germany; 2 Department of General, Abdominal and Transplantation Surgery, University Hospital Heidelberg, Heidelberg, Germany; 3 Medical and Biological Informatics, German Cancer Research Center, Heidelberg, Germany; 4 Department of Radiology, German Cancer Research Center (dkfz) Heidelberg, INF 280, Heidelberg, Germany; 5 Department of Anesthesiology, University Hospital Heidelberg, Heidelberg, Germany; 6 Clinic for Radiology, Minimally-invasive Therapies and Nuclear Medicine, SLK Kliniken Heilbronn GmbH, Heilbronn, Germany

**Keywords:** Irreversible electroporation, Liver, CT 3d rendering, Segmentation, Chebyshev center

## Abstract

**Background:**

Size and shape of the treatment zone after Irreversible electroporation (IRE) can be difficult to depict due to the use of multiple applicators with complex spatial configuration. Exact geometrical definition of the treatment zone, however, is mandatory for acute treatment control since incomplete tumor coverage results in limited oncological outcome. In this study, the “Chebyshev Center Concept” was introduced for CT 3d rendering to assess size and position of the maximum treatable tumor at a specific safety margin.

**Methods:**

In seven pig livers, three different IRE protocols were applied to create treatment zones of different size and shape: Protocol 1 (n = 5 IREs), Protocol 2 (n = 5 IREs), and Protocol 3 (n = 5 IREs). Contrast-enhanced CT was used to assess the treatment zones. Technique A consisted of a semi-automated software prototype for CT 3d rendering with the “Chebyshev Center Concept” implemented (the “Chebyshev Center” is the center of the largest inscribed sphere within the treatment zone) with automated definition of parameters for size, shape and position. Technique B consisted of standard CT 3d analysis with manual definition of the same parameters but position.

**Results:**

For Protocol 1 and 2, short diameter of the treatment zone and diameter of the largest inscribed sphere within the treatment zone were not significantly different between Technique A and B. For Protocol 3, short diameter of the treatment zone and diameter of the largest inscribed sphere within the treatment zone were significantly smaller for Technique A compared with Technique B (41.1 ± 13.1 mm versus 53.8 ± 1.1 mm and 39.0 ± 8.4 mm versus 53.8 ± 1.1 mm; p < 0.05 and p < 0.01). For Protocol 1, 2 and 3, sphericity of the treatment zone was significantly larger for Technique A compared with B.

**Conclusions:**

Regarding size and shape of the treatment zone after IRE, CT 3d rendering with the “Chebyshev Center Concept” implemented provides significantly different results compared with standard CT 3d analysis. Since the latter overestimates the size of the treatment zone, the “Chebyshev Center Concept” could be used for a more objective acute treatment control.

## Background

Focal tumor ablation is an accepted option for the treatment of primary and secondary malignant liver tumors. Irreversible electroporation (IRE) was introduced as a non-thermal technique for tissue destruction. After local application of high-voltage electrical pulses of microsecond duration, homogeneous areas of non-viable cells are induced [1]. In first clinical studies, IRE demonstrates promising results for the treatment of malignant liver lesions [2-4]. The routine use of IRE, however, is still not established [5,6]. Whereas radiofrequency ablation and microwave ablation induce cell death via thermal damage, the exact mechanisms for IRE are not entirely understood [7]. Nonetheless, IRE is attributed with potential advantages compared with thermal ablation (e.g. reduced collateral damage and insignificance of the heat-sink effect) [5]. Those advantages can be explained with the relative resistance of low lipid containing structures (e.g. extracellular matrix and endothelial cells) to the electrical pulses while high lipid containing structures (e.g. tumor cells) can be destroyed completely [8,9]. Accordingly, IRE should be a promising alternative especially if tumors are located near vulnerable structures.

As with thermal ablation, the treatment zone after IRE must cover the entire tumor in addition to a safety margin. As demonstrated by Wang et al., the size of the safety margin plays a key role for the oncological success of focal tumor ablation [10]. After radiofrequency ablation of colorectal liver metastases, they found that a safety margin uniformly larger than 5 mm, defined with post-interventional contrast-enhanced CT, is associated with better local tumor control. For the combination of optimal oncological outcome with liver-sparing tumor ablation, which is relevant for the minimization of procedure-related complications, another prerequisite is mandatory: congruency of tumor center and coagulation center [11]. Currently, there exists no clinically relevant software that can be used to determine whether the treated tumor and the intended safety margin are covered completely by the treatment zone. Standard CT 3d analysis of the treatment zone after focal tumor ablation consists of measurements based on standard CT image planes (e.g. axial), without meeting the clinical requirements of interventional radiologists in terms of objective treatment control. A major drawback of currently available 3d software is suboptimal assessment of the exact geometry of the treatment zone in relation to the tumor extent. For example indentations limiting the expansion of the treatment zone need to be assessed since those are likely to represent the site of incomplete tumor destruction and local recurrence. Since IRE can be performed with lots of different protocols (e.g. up to six applicators with a tip exposure of up to 40 mm) all affecting significantly size and shape of the treatment zone, the exact geometrical assessment is mandatory to further improve the procedural success. This issue was published by Adeyanju et al., who demonstrated that IRE protocols impact not only the extent of the treatment zone but also the maximum treatable tumor size [12].

With this background, the objective of our study was defined: to introduce the “Chebyshev Center Concept” for CT 3d rendering to assess size and position of the maximum treatable tumor size at a specific safety margin after IRE in a pig liver model, and to demonstrate better performance compared with standard CT 3d analysis. For our study, the “Chebyshev Center” of the treatment zone is the center of the largest inscribed sphere within the treatment zone. In geometry, the largest inscribed sphere is the sphere that bounds the edges of a three-dimensional body in such a manner that there is no other sphere that lies completely within that three-dimensional body and at the same time has a larger diameter than the largest inscribed sphere. The three-dimensional body corresponds to the treatment zone, and the largest inscribed sphere is the largest sphere that is covered completely by the treatment zone. The “Chebyshev Center Concept” can be used to quantify and visualize diameter and position of the largest inscribed sphere within the treatment zone after focal tumor ablation. This sphere is relevant since it allows the definition of the maximum treatable tumor size at a specific safety margin. Furthermore, the “Chebyshev Center Concept” is applicable to determine whether tumor and intended safety margin is located within the treatment zone (or in other words whether the concrete treatment zone is adequate for a specific tumor extent).

### Methods

The experiments were performed in accordance with the “Guide for the Care and Use of Laboratory Animals”. “State Animal Care and Ethics Committee” approval was obtained.

### Animal preparation

Seven healthy landrace pigs with a body weight between 35 and 41 kg were sedated with an intramuscular cocktail consisting of 10 mg ketamine, 6 mg azaperone and 0.4mg midazolam per kg body weight. Peripheral and central venous catheters were installed. After intubation, anesthesia was maintained with isoflurane. Intravenous bolus injections with pancuronium were used to induce and maintain muscle relaxation. A continuous 4-lead electrocardiogram was performed throughout the procedure. Before and immediately after IRE, contrast-enhanced CT was performed with a 128-slice multi-detector row CT scanner (Somatom Definition Flash; Siemens Medical Solutions, Forchheim, Germany). The CT protocol consisted of a non-enhanced phase, and after intravenous injection of 70 ml of iodinated contrast material, arterial (delay of 5 s after reaching the trigger threshold of 100 HU), venous (delay of 50 s) and late (delay of 180 s) phases followed. Image reconstructions included axial image planes with a slice thickness of 1 mm and an overlap of 0.5 mm. The CT scan immediately after IRE was intended as acute treatment control according to best clinical practice. Animals were sacrificed subsequently.

### IRE procedure

IRE was performed with a commercial generator (NanoKnife™ Electroporator; AngioDynamics® Inc., Queensbury, USA). Electrocardiogram synchronization was used to prevent cardiac arrhythmias. Commercial monopolar 19G applicators (NanoKnife™ Applicator; AngioDynamics® Inc., Queensbury, USA) served for local pulse application. A total of 15 IREs were carried out. Three different IRE protocols were used to obtain different extents of the treatment zone: Protocol 1 (three applicators, tip exposure of 20 mm, distance between pairs of applicators of 15 mm, pulse number of 90, pulse length of 90 μs, and electric field of 1500 V/cm; n = 5 IREs), Protocol 2 (three applicators, tip exposure of 25 mm, distance between pairs of applicators of 20 mm, pulse number of 90, pulse length of 90 μs, and electric field of 1500 V/cm; n = 5 IREs), and Protocol 3 (six applicators, tip exposure of 30 mm, distance between pairs of applicators of 15 mm, pulse number of 70, pulse length of 90 μs, and electric field of 1400 V/cm; n = 5 IREs). Based on the information of the manufacturer as well as on findings of published and own IRE experience, those protocols allow predicting the size of the treatment zone, with treatment zones between 1 and 5 cm. In this context, the different mechanisms of tissue destruction between IRE and thermal ablation are important to mention. In thermal ablation, heat expands from the tip of an applicator to the periphery, and power output and ablation time are major predictors for size and shape of the coagulation zone. On the contrary, size and shape of the treatment zone after IRE is determined by number and spatial configuration of applicators, tip exposure, pulse number, pulse length as well as electric field. The treatment zones expand between the different pairs of applicators, resulting finally in one overlapping treatment zone. According to our treatment plan, applicators were positioned standardized across all animals. In one pig, two IREs according to Protocol 1 were performed in the right liver and one IRE according to Protocol 2 was performed in the left liver. In another pig, one IRE according to Protocol 1 was performed in the left liver and one IRE according to Protocol 2 was performed in the right liver. In two other pigs, one IRE according to Protocol 1 was performed in the left liver and one IRE according to Protocol 3 was performed in the right liver. In the three remaining pigs, one IRE according to Protocol 2 was performed in the left liver and one IRE according to Protocol 3 was performed in the right liver. Consequently, in six pigs a maximum of 2 IREs per pig was realized, and in one pig a maximum of three IREs was realized. This proceeding avoided overlap and interaction between the IREs in the same liver (e.g. impact of microvascularization resulting in inaccurate CT enhancement patterns after application of intravenous contrast material). All applicators were positioned step-by-step in parallel fashion under CT guidance. The correct applicator configuration according to the treatment plan was confirmed with a non-enhanced CT scan using multi-planar image planes.

### Analysis of size and shape of the treatment zone

**Figure 1:**
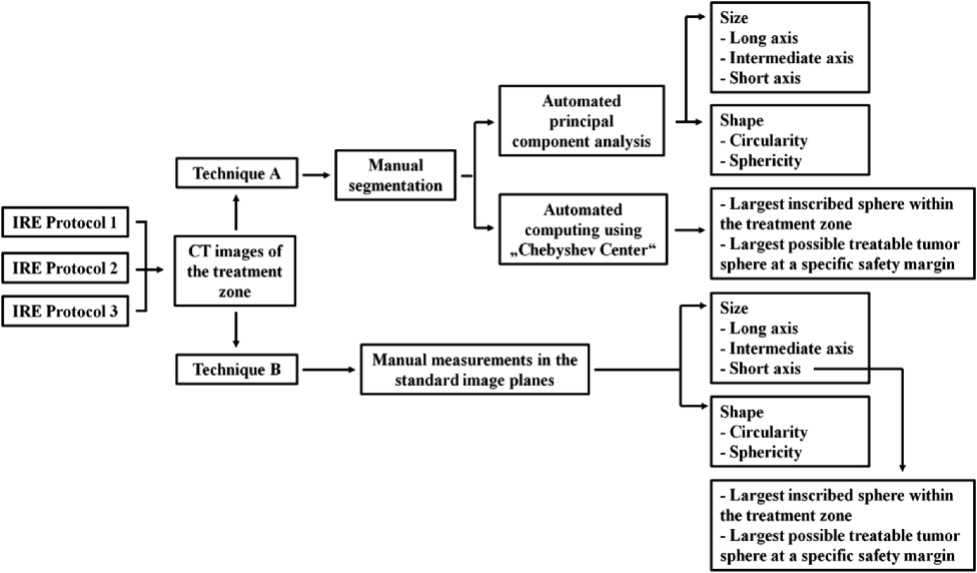
Flowchart for illustration of the analysis of the treatment zone.

The image data were extracted from our institutional prospective digital database (GE Centricity 4.1, GE Healthcare, Barrington, USA) and analyzed on a PC applying the software described in this study. Two techniques for the analysis of the treatment zone were compared: Technique A versus Technique B (not to be confused with the different IRE Protocols) (Figure 1). According to other publications, the treatment zone after IRE was defined as the hypodense area with sharp demarcation on contrast-enhanced CT images of the venous phase [13,14].

### Technique A

**Figure 2:**
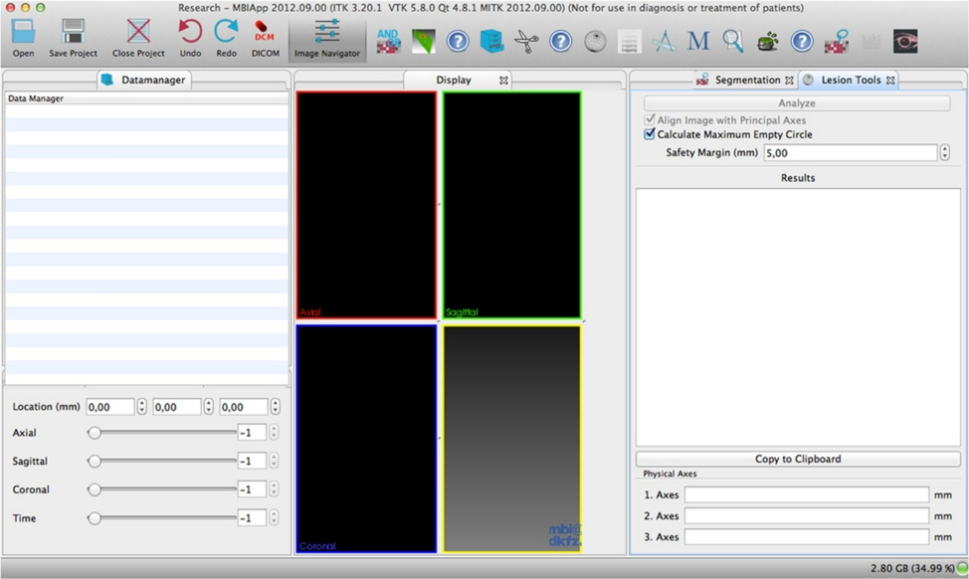
**Technique A (Semi-automated Software Prototype for CT 3d Rendering with the “Chebyshev Center Concept” implemented) - User Interface.** The MITK user interface consists of three work columns: “data manager” in the left column, “display” in the middle column, and post-processing tools (“Segmentation” and “Lesion Tools” in this case) in the right column. Images in different formats (e.g. DICOM or JPEG) can be uploaded and scrolled in the “data manager” column. Multi-planar image planes are visualized in the “display” column. In the post-processing tool column, the treatment zone can be outlined manually by means of cursor (in the sense of a segmentation) applying “Segmentation”. Then, applying “Lesion Tools”, long, intermediate and short diameter, circularity and sphericity as well as the diameter of the largest inscribed sphere within the treatment zone and the diameter of the largest possible treatable tumor sphere at a defined safety margin (in this case 5mm) inclusive of the barycenter offset can be calculated automatically.

Technique A consisted of a semi-automated software prototype for CT 3d rendering with the “Chebyshev Center Concept” implemented (MITK freeware “Geometric evaluation of ablations” http://www.mitk.org/AblationEvaluation; German Cancer Research Center (dkfz) Heidelberg, Heidelberg, Germany). Axial image planes with a slice thickness of 1 mm and an overlap of 0.5 mm were uploaded (Figure 2). Treatment zones were outlined manually by means of cursor in the sense of segmentation. After segmentation, parameters for size and shape (long, intermediate and short diameter, circularity and sphericity as well as the diameter of the largest inscribed sphere within the treatment zone and the diameter of the largest possible treatable tumor sphere for both applying the “Chebyshev Center”) were obtained automatically. The geometrical center of the treatment zone (from now on the barycenter) was determined automatically by using a principal component analysis. The diameter of the treatment zone through the barycenter in direction of the eigenvector with the largest principal component was defined as the long diameter. The diameter of the treatment zone perpendicular to the long diameter and through the barycenter in direction of the second largest principal component eigenvector was defined as the intermediate diameter. The third diameter of the treatment zone perpendicular to the long and intermediate diameter and through the barycenter was defined as the short diameter. Consequently, the barycenter is the intersection point of long, intermediate and short diameter of the treatment zone. Circularity and sphericity of the treatment zone are measures to describe the shape of the treatment zone applying long, intermediate and/or short diameter [15-17]:

(1) Circularity of the treatment zone = short diameter/long diameter (a number of “1” indicates a perfect roundness) and

(2) Sphericity of the treatment zone = long diameter/((intermediate diameter + short diameter)/2) (a number of “1” indicates a perfect roundness).

The largest inscribed sphere within the treatment zone was defined automatically. As mentioned above, the “Chebyshev Center” is the center of this sphere, and can be applied for the determination of diameter and position (barycenter offset) of the largest inscribed sphere within the treatment zone. Thereby, the barycenter offset is the distance between the barycenter and the “Chebyshev Center”. The diameter of the largest possible treatable tumor sphere was defined automatically also. This sphere has the same center as the largest inscribed sphere within the treatment zone (“Chebyshev Center”). Moreover, the largest possible treatable tumor sphere has a diameter 10 mm smaller as the short diameter (corresponding to a uniform safety margin of 5 mm).

### Technique B

**Figure 3:**
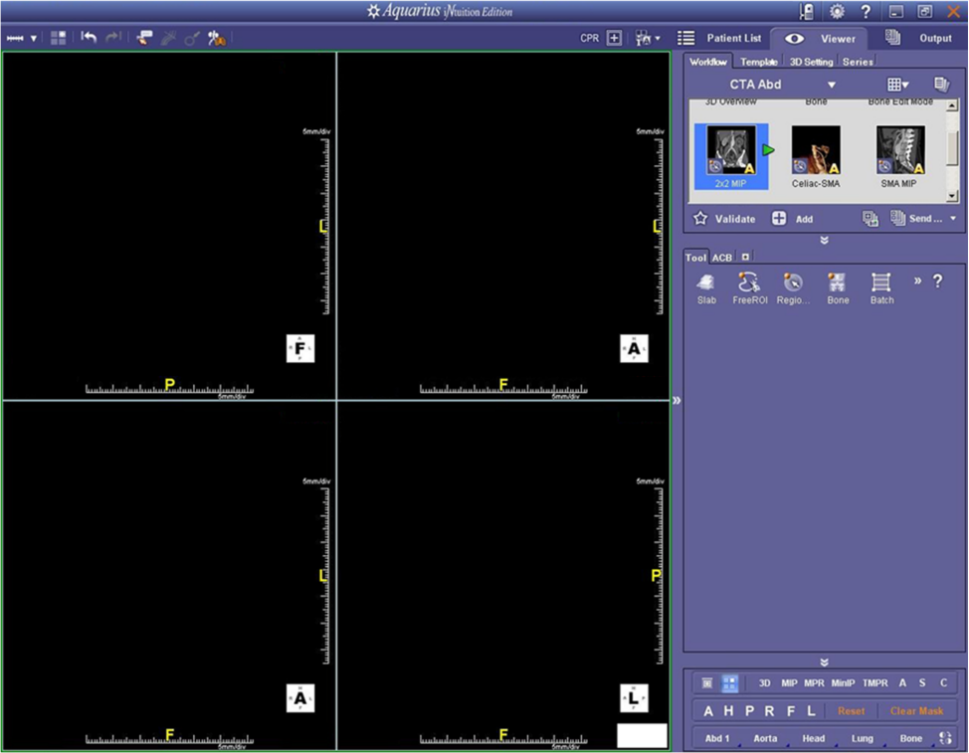
**Technique B (Standard CT 3d Analysis) - User Interface.** This user interface is clinically established, and provides the possibility to measure manually diameters on axial, coronal and sagittal image planes (CTA abdomen workflow, multi-planar mode).

Technique B consisted of standard CT 3d analysis without specific software assistance (such as principal component analysis). Axial image planes with a slice thickness of 1 mm and an overlap of 0.5 mm were uploaded in the “CTA Abdomen” workflow on a commercial work station (TeraRecon, INC., Aquarius, iNtuition™ Edition, Ver. 4.4.4.23.771, San Mateo, USA) (Figure 3). Applying the multi-planar mode, the absolute longest diameter of the treatment zone was measured manually on axial image planes. Perpendicular to this diameter, the longest diameter of the treatment zone was measured manually on axial image planes. On coronal image planes, the longest craniocaudal diameter of the treatment zone was measured manually. This proceeding is in line with current clinical practice for treatment control after radiofrequency ablation [15-17]. The three measured diameters were ordered by size, and defined as long, intermediate and short diameter of the treatment zone. Circularity and sphericity of the treatment zone were calculated according to equations (1) and (2). The diameter of the largest inscribed sphere within the treatment zone was defined equal to the short diameter. The diameter of the largest possible treatable tumor sphere was defined using a diameter 10 mm smaller as the diameter of the largest inscribed sphere within the treatment zone. For Technique B, the barycenter offset was not assessable.

### Differences between technique A and technique B

To quantify the differences between Technique A and Technique B regarding the largest inscribed sphere within the treatment zone, the difference ratio was calculated:

(3) Difference ratio = Diameter of the largest inscribed sphere within the treatment zone_Technique A_ - Diameter of the largest inscribed sphere within the treatment zone_Technique B_.

The difference ratio indicates the absolute difference between Technique A and Technique B. A negative difference ratio means that the parameter for Technique A is smaller than for Technique B (or in other words that standard CT 3d analysis overestimates the extent of the treatment zone), whereas a positive difference ratio means that the parameter for Technique A is larger than for Technique B (or in other words that standard CT 3d analysis underestimates the extent of the treatment zone).

### Statistics

Prism software (Version 6.00, GraphPad Software, LaJolla, USA) was used. Quantitative data were presented as mean ± standard deviation, and range. To evaluate statistical differences between Technique A and Technique B, the Wilcoxon signed-rank test was used. To describe statistical differences between IRE Protocol 1, Protocol 2 and Protocol 3, the Kruskal-Wallis test was applied. P < 0.05 was defined as the level of significance.

## Results and discussion

The mean duration of the IRE procedures per pig was 37 ± 12 min (25-70 min).

### Size of the treatment zone

**Figure 4:**
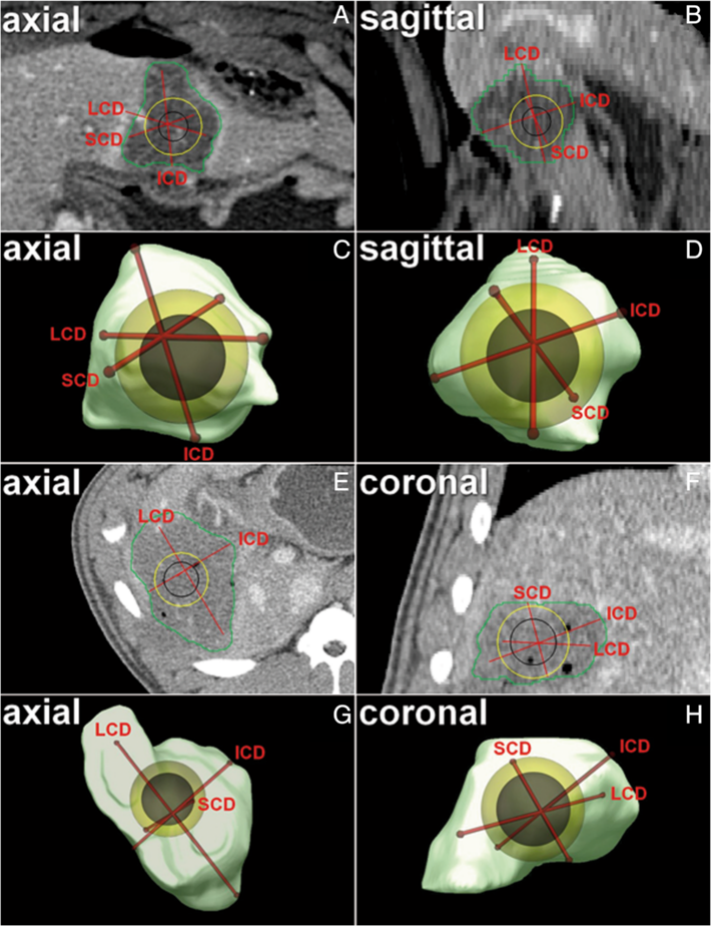
**Technique A (Semi-automated Software Prototype for CT 3d Rendering with the “Chebyshev Center Concept” implemented) - Image Example. A-D** Axial image plane **(A)** and sagittal image plane **(B)** as well as corresponding volume rendering **(C, D)** – IRE Protocol 2. **E-H** Axial image plane **(E)** and coronal image plane **(F)** as well as corresponding volume rendering **(G, H)** – IRE Protocol 3. Note: after manual segmentation of the treatment zone (green), long, intermediate and short diameter (LCD, ICD and SCD) as well as the largest inscribed sphere within the treatment zone (yellow) and the largest possible treatable tumor sphere (black) were defined automatically (in Figure 4E, SCD is not indicated since its craniocaudal course). The barycenter offset is the distance between the barycenter of the treatment zone (intersection point of long, intermediate and short diameter) and the “Chebyshev Center” (center of the largest inscribed sphere within the treatment zone = center of the largest possible treatable tumor sphere). Observe the conspicuous eccentricity of the “Chebyshev Center” within the treatment zone (which is quantified by the barycenter offset) on standard image planes (B and E) which is not assessable with standard CT 3d analysis (Figure 5). Visualization and quantification of size and position of the largest inscribed sphere within the treatment zone (as well as of the largest possible treatable tumor sphere) can be relevant for acute treatment control after IRE (e.g. confirmation of the intended safety margin size).

**Figure 5:**
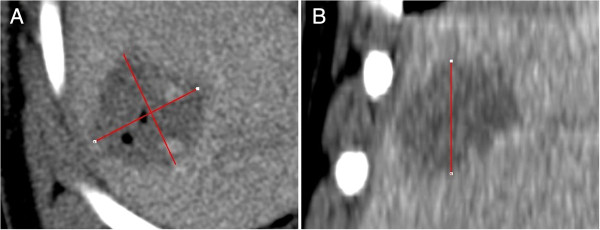
**Technique B (Standard CT 3d Analysis) - Image Example. A**, **B** axial image plane **(A)** as well as coronal image plane **(B)** – IRE Protocol 1. Note: in the axial image plane, the absolute longest diameter of the treatment zone was measured, and perpendicular to this parameter, the longest diameter of the treatment zone was determined. In the coronal image plane, the longest craniocaudal diameter of the treatment zone was determined. These three diameters were ordered by size and defined as long, intermediate and short diameter. The diameter of the largest inscribed sphere within the treatment zone was defined equal to the short diameter. The barycenter offset is not assessable applying this approach.

**Figure 6:**
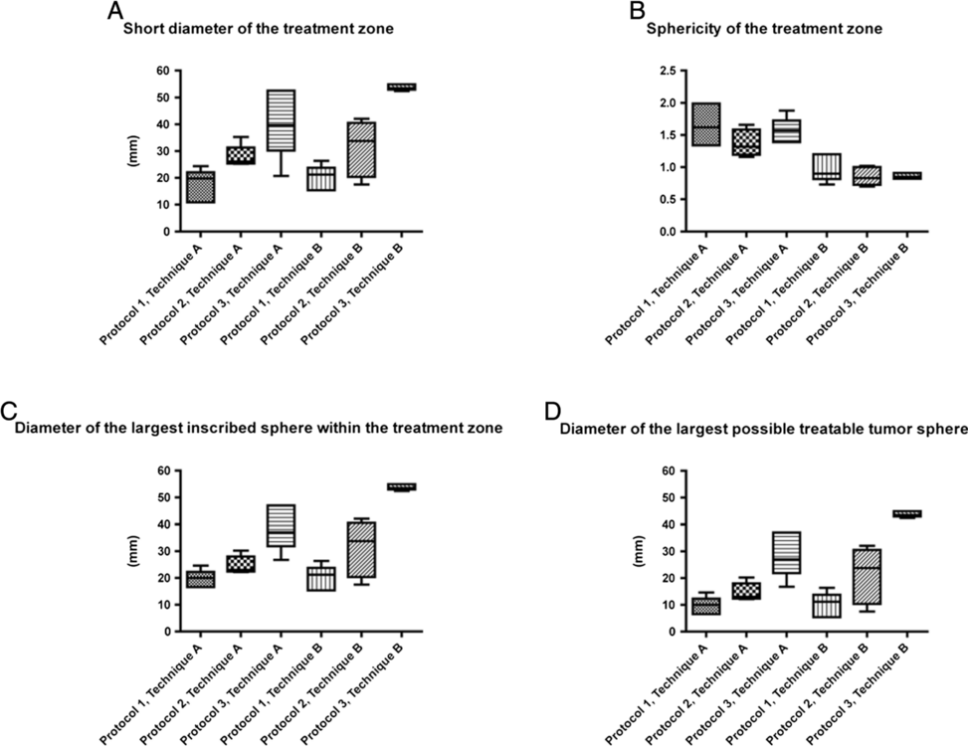
**Summary of the most relevant Parameters for Size and Shape. A** Short diameter of the treatment zone. Note: For Protocol 1 and 2, there was a trend for a smaller short diameter for Technique A, respectively. For Protocol 3, short diameter was significantly smaller for Technique A compared with Technique B (p < 0.05). Short diameter for Technique A and Technique B was significantly different between Protocol 1, 2 and 3 (p < 0.01 and p < 0.005, respectively). **B** Sphericity of the treatment zone. Note: For Protocol 1, 2 and 3, sphericity was significantly larger for Technique A compared with Technique B, respectively (p < 0.01, p < 0.01 and p < 0.01, respectively). Sphericity for Technique A and B was not significantly different between Protocol 1, 2 and 3. **C** Diameter of the largest inscribed sphere within the treatment zone. Note: For Protocol 2, there was a trend for a smaller diameter of the largest inscribed sphere within the treatment zone for Technique A. For Protocol 3, the diameter of the largest inscribed sphere within the treatment zone was significantly smaller for Technique A compared with Technique B (p < 0.01). The diameter of the largest inscribed sphere within the treatment zone for Technique A and B was significantly different between Protocol 1, 2 and 3 (p < 0.005 and p < 0.005, respectively). **D** Diameter of the largest possible treatable tumor sphere. Note: For Protocol 3, the diameter of the largest possible treatable tumor sphere was significantly smaller for Technique A compared with Technique B (p < 0.01). The diameter of the largest inscribed sphere within the treatment zone for Technique A and Technique B was significantly different between Protocol 1, 2 and 3 (p < 0.005 and p < 0.005, respectively).

Detailed data are presented in Table 1 (Figures 4, 5 and 6). For Protocol 1 and Protocol 2, long, intermediate and short diameters were not significantly different between Technique A and Technique B, respectively. There was a trend for a larger long diameter for Technique A for Protocol 1 as well as a trend for smaller short diameter for Technique A for Protocol 1 and Protocol 2, respectively. For Protocol 3, long and intermediate diameters were not significantly different between Technique A and Technique B, respectively. There was a trend for a larger long diameter for Technique A for Protocol 3. For Protocol 3, the short diameter was significantly smaller for Technique A compared with Technique B (41.1 ± 13.1 mm versus 53.8 ± 1.1 mm; p < 0.05). All parameters for Technique A and Technique B were significantly different between Protocol 1, Protocol 2 and Protocol 3, respectively.

**Table 1:** Size of the treatment zone

	**Long diameter (mm)**		**Intermediate diameter (mm)**		**Short diameter (mm)**	
	Technique A^1^	Technique B^2^	Technique A^1^	Technique B^2^	Technique A^1^	Technique B^2^
Protocol 1^3^	42.1 ± 3.2^#,*^	37.5 ± 10.2^#,**^	30.1 ± 3.2^+,***^	27.9 ± 5.5^+,****^	17.2 ± 6.0°^,*****^	19.9 ± 4.6°^,******^
	(39.3 - 47.2)	(29.4 - 48.6)	(26.6 - 33.7)	(22.0 - 32.7)	(10.9 - 24.4)	(15.4 - 26.4)
Protocol 2^4^	43.7 ± 8.6^##,*^	43.8 ± 3.9^##,**^	35.7 ± 8.2^++,***^	37.1 ± 6.2^++,****^	27.9 ± 4.2°°^,*****^	31.1 ± 10.5°°^,******^
	(30.4 – 52.8)	(40.1 - 50.2)	(26.1 – 44.8)	(30.6 - 46.0)	(25.3 - 35.3)	(17.5 - 42.1)
Protocol 3^5^	74.5 ± 6.1^###,*^	71.0 ± 3.4^###,**^	56.3 ± 5.3^+++,***^	56.3 ± 2.9^+++,****^	41.1 ± 13.1°°°^,*****^	53.8 ± 1.1°°°^,******^
	(63.7 - 78.1)	(67.3 - 75.1)	(47.2 - 60.1)	(52.7 - 59.2)	(20.7 - 52.6)	(52.4 - 54.9)

^1^semi-automated software prototype for CT 3d rendering with the “Chebyshev Center Concept” implemented;

^2^standard CT 3d analysis;

^3^Protocol 1 with n = 5 IREs (three applicators, tip exposure of 20 mm, distance between pairs of applicators of 15 mm, pulse number of 90, pulse length of 90 μs, and electric field of 1500 V/cm);

^4^Protocol 2 with n = 5 IREs (three applicators, tip exposure of 25 mm, distance between pairs of applicators of 20 mm, pulse number of 90, pulse length of 90 μs, and electric field of 1500 V/cm);

^5^Protocol 3 with n = 5 IREs (six applicators, tip exposure of 30 mm, distance between pairs of applicators of 15 mm, pulse number of 70, pulse length of 90 μs, and electric field of 1400 V/cm);

statistical differences between Technique A and Technique B were analyzed with the Wilcoxon signed-rank test: ^#^p > 0.05; ^##^p > 0.05; ^###^p > 0.05; ^+^p > 0.05; ^++^p > 0.05; ^+++^p > 0.05; °p > 0.05; °°p > 0.05; °°°p < 0.05;

statistical differences between Protocol 1, Protocol 2 and Protocol 3 were analyzed with the non-parametric Kruskal-Wallis test: ^*^p < 0.01; ^**^p < 0.01; ^***^p < 0.01; ^****^p < 0.005; ^*****^p < 0.01; ^******^p < 0.005.

### Shape of the treatment zone

Detailed data are presented in Table 2 (Figures 4, 5 and 6). For Protocol 1 and Protocol 2, circularity was not significantly different between Technique A and Technique B, respectively. Sphericity was significantly larger for Technique A compared with Technique B for Protocol 1 and Protocol 2, respectively (1.7 ± 0.3 versus 1.0 ± 0.2 and 1.4 ± 0.2 versus 0.9 ± 0.1; p < 0.01 and p < 0.01, respectively). For Protocol 3, circularity and sphericity were significantly different between Technique A and Technique B, respectively (0.5 ± 0.2 versus 0.8 ± 0.1 and 1.6 ± 0.2 versus 0.9 ± 0.1; p < 0.01 and p < 0.01, respectively). Circularity and sphericity for Technique A and Technique B were not significantly different between Protocol 1, Protocol 2 and Protocol 3, respectively.

**Table 2:** Shape of the treatment zone

	**Circularity**		**Sphericity**	
	Technique A^1^	Technique B^2^	Technique A^1^	Technique B^2^
Protocol 1^3^	0.4 ± 0.1^#,*^	0.6 ± 0.3^#,**^	1.7 ± 0.3^+,***^	1.0 ± 0.2^+,****^
	(0.3 - 0.5)	(0.3 - 0.8)	(1.3 - 2.0)	(0.7 - 1.2)
Protocol 2^4^	0.7 ± 0.1^##,*^	0.7 ± 0.2^##,**^	1.4 ± 0.2^++,***^	0.9 ± 0.1^++,****^
	(0.5 - 0.9)	(0.4 - 0.9)	(1.2 - 1.7)	(0.7 - 1.0)
Protocol 3^5^	0.5 ± 0.2^###,*^	0.8 ± 0.1^###,**^	1.6 ± 0.2^+++,***^	0.9 ± 0.1^+++,****^
	(0.3 - 0.7)	(0.7 - 0.8)	(1.4 - 1.9)	(0.8 - 0.9)

^1^semi-automated software prototype for CT 3d rendering with the “Chebyshev Center Concept” implemented;

^2^standard CT 3d analysis;

^3^Protocol 1 with n = 5 IREs (three applicators, tip exposure of 20 mm, distance between pairs of applicators of 15 mm, pulse number of 90, pulse length of 90 μs, and electric field of 1500 V/cm);

^4^Protocol 2 with n = 5 IREs (three applicators, tip exposure of 25 mm, distance between pairs of applicators of 20 mm, pulse number of 90, pulse length of 90 μs, and electric field of 1500 V/cm);

^5^Protocol 3 with n = 5 IREs (six applicators, tip exposure of 30 mm, distance between pairs of applicators of 15 mm, pulse number of 70, pulse length of 90 μs, and electric field of 1400 V/cm);

statistical differences between Technique A and Technique B were analyzed with the Wilcoxon signed-rank test: ^#^p > 0.05; ^##^p > 0.05; ^###^p < 0.01; ^+^p < 0.01; ^++^p < 0.01; ^+++^p < 0.01;

statistical differences between Protocol 1, Protocol 2 and Protocol 3 were analyzed with non-parametric Kruskal-Wallis test: ^*^p > 0.05; ^**^p > 0.05; ^***^p > 0.05; ^****^p > 0.05.

### Diameter and position of the largest inscribed sphere within the treatment zone

Detailed data are presented in Table 3 (Figures 4 and 6). For Protocol 1 and Protocol 2, the diameter of the largest inscribed sphere within the treatment zone as well as the diameter of the largest possible treatable tumor sphere were not significantly different between Technique A and Technique B, respectively. There was a trend for a smaller diameter of the largest inscribed sphere within the treatment zone as well as a trend for a smaller diameter of the largest possible treatable tumor sphere for Technique A for Protocol 2, respectively. For Protocol 3, the diameter of the largest inscribed sphere within the treatment zone as well as the diameter of the largest possible treatable tumor sphere were significantly different between Technique A and Technique B, respectively (39.0 ± 8.4 mm versus 53.8 ± 1.1 mm and 29.0 ± 8.4 mm versus 43.8 ± 1.1 mm; p < 0.01 and p < 0.01, respectively). The barycenter offset was not assessable for Technique B. All parameters but the barycenter offset for Technique A and Technique B were significantly different between Protocol 1, Protocol 2 and Protocol 3, respectively.

**Table 3:** Diameter and position of the largest inscribed sphere within the treatment zone as well as of the largest possible treatable tumor sphere

	**Diameter of the largest inscribed sphere within the treatment zone (mm)**		**Diameter of the largest possible treatable tumor sphere (mm)**		**Barycenter offset**^**1**^**(mm)**	
	Technique A^2^	Technique B^3^	Technique A^2^	Technique B^3^	Technique A^2^	Technique B^3^
Protocol 1^4^	19.6 ± 3.3^#,*^	19.9 ± 4.6^#,**^	9.6 ± 3.3^+,***^	9.9 ± 4.6^+,****^	9.1 ± 2.1^*****^	n.a.
	(16.6 - 24.6)	(15.4 - 26.4)	(6.6 - 14.6)	(5.4 - 16.4)	(5.4 - 10.6)	
Protocol 2^5^	24.8 ± 3.4^##,*^	31.1 ± 10.5^##,**^	14.8 ± 3.4^++,***^	21.1 ± 10.5^++,****^	5.4 ± 3.6^*****^	n.a.
	(22.2 - 30.2)	(17.5 - 42.1)	(12.2 - 20.2)	(7.5 - 32.1)	(1.2 - 10.1)	
Protocol 3^6^	39.0 ± 8.4^###,*^	53.8 ±1.1^###,**^	29.0 ± 8.4^+++,***^	43.8 ±1.1^+++,****^	5.9 ± 3.8^*****^	n.a.
	(26.8 - 47.0)	(52.4 - 54.9)	(16.8 - 37.0)	(42.4 - 44.9)	(2.4 - 11.7)	

^1^distance between the barycenter of the treatment zone (intersection point of long, intermediate and short diameter) and the “Chebyshev Center”

^2^semi-automated software prototype for CT 3d rendering with the “Chebyshev Center Concept” implemented;

^3^standard CT 3d analysis;

^4^Protocol 1 with n = 5 IREs (three applicators, tip exposure of 20 mm, distance between pairs of applicators of 15 mm, pulse number of 90, pulse length of 90 μs, and electric field of 1500 V/cm);

^5^Protocol 2 with n = 5 IREs (three applicators, tip exposure of 25 mm, distance between pairs of applicators of 20 mm, pulse number of 90, pulse length of 90 μs, and electric field of 1500 V/cm);

^6^Protocol 3 with n = 5 IREs (six applicators, tip exposure of 30 mm, distance between pairs of applicators of 15 mm, pulse number of 70, pulse length of 90 μs, and electric field of 1400 V/cm);

statistical differences between Technique A and Technique B were analyzed with the Wilcoxon signed-rank test: ^#^p > 0.05; ^##^p > 0.05; ^###^p < 0.01; ^+^ p > 0.05; ^++^ p > 0.05; ^+++^p < 0.01;

statistical differences between Protocol 1, Protocol 2 and Protocol 3 were analyzed with the Kruskal-Wallis test: ^*^p < 0.005; ^**^p < 0.005; ^***^p < 0.005; ^****^p < 0.005; ^*****^p > 0.05;

n.a. not assessable.

### Differences between technique A and technique B

For Protocol 1, Protocol 2 and Protocol 3, the difference ratio was negative (-0.4 ± 1.5 mm (-1.8-1.2 mm), -6.4 ± 8.7 mm (-16.3-5.1 mm) and -14.9 ± 7.4 mm (-25.6--7.9 mm), respectively). Thereby, significant differences existed between Protocol 1, Protocol 2 and Protocol 3 (p < 0.05).

## Discussion

In this in-vivo pig liver study, significant differences regarding the geometry of the treatment zone after IRE exist when CT 3d rendering with the “Chebyshev Center Concept” implemented (Technique A) is compared with standard CT 3d analysis (Technique B). In summary, the diameter of the largest inscribed sphere within the treatment zone and the diameter of the largest possible treatable tumor sphere either were identical (for IRE Protocol 1), tended to be smaller (for IRE Protocol 2), or were significantly smaller (for IRE Protocol 3) for Technique A compared with Technique B. For all IRE protocols, sphericity was significantly larger for Technique A compared with Technique B indicating rounder treatment zones for the latter. On contrary to Technique B, the position of the largest inscribed sphere within the treatment zone as well as the position of the largest possible treatable tumor sphere within the treatment zone could be quantified and visualized for Technique A.

Standard techniques for the evaluation of the treatment zone after focal tumor ablation are manual measurements of perpendicular diameters on axial, coronal and/or sagittal image planes [18,19]. With such a proceeding, however, the geometry of the treatment zone might be assessed not as precise as necessary. As it has been demonstrated for liver tumors, 3d segmentation showed a better depiction of tumor size and shape compared with conventional image analysis [20,21]. For hepatocellular carcinoma, Galizia et al. found that the maximum tumor diameter is significantly different between 3d analysis and control (32 ± 9 mm vs. 35 ± 12 mm; p < 0.001) [20]. Rothe et al. analyzed 102 liver metastases in 45 patients, and found significantly lower volumes for 3d analysis compared with 2d analysis, with relative differences up to 41.1% [21]. For focal tumor ablation, comparable data is very rare. In a work published by Elhawary et al., 3d volumetric non-rigid image registration during cryoablation could improve the ablation procedure relating to planning, targeting and evaluation of tumor coverage [22]. In another study, segmentation with image fusion results in a more accurate treatment control after radiofrequency ablation [23]. Accordingly, the depiction of the treatment zone after IRE should be more exact for Technique A compared with Technique B since Technique A determines automatically the 3 perpendicular diameters after 3d segmentation without being limited to analyses in the standard image planes. Technique A allows not only assessment of the objective size of the treatment zone (especially definition of the short diameter, which is of paramount importance for the oncological success) but also of the objective shape of the treatment zone (which can be relevant to reduce procedure-related complications such as protection of viable structures) [19]. Technique A is independent of the tumor orientation with respect to the standard imaging planes. It is a reproducible automatic method based on a standard mathematical analysis (principal component analysis) of geometric shapes and also suitable for automatic comparisons of geometric features between imaging sessions. Our data suggest that the larger the treatment zone is, the larger the difference regarding size between Technique A and Technique B is. Regarding shape, Technique A and Technique B showed significantly different results for all IRE protocols. Both issues might emphasize the need for specific 3d rendering techniques for focal tumor ablation if one intends an optimal procedural outcome [24].

The spatial positioning of the multiple IRE applicators lead to complex treatment zones regarding shape, indentations and skip lesions compared with the rather predictable ellipsoid treatment zones after thermal ablation created with one straight needle design applicator [11,12,18]. Technique A can be used for a practicable and precise analysis of complex treatment zones after IRE. Per definition, the “Chebyshev Center” is the center of the largest inscribed sphere within a three dimensional body. In our study, this concept is used to define diameter and position of the largest inscribed sphere within the treatment zone. Technique A aligns automatically the largest inscribed sphere within the treatment zone, and calculates the barycenter offset. Due to the fact that the diameter of the largest possible treatable tumor sphere is smaller than the diameter of the largest inscribed sphere within the treatment zone by twice the safety margin, and since both spheres have the same center (the “Chebyschev Center”), the precise location of the largest possible treatable tumor sphere can be easily defined. For Technique B, the diameter of the largest possible treatable tumor sphere is determined with the short diameter, and the barycenter offset is not assessable. The presented differences between Technique A and Technique B result since only Technique A takes into account the exact geometry of the treatment zone including all shape irregularities (e.g. indentations). This means, if Technique B is applied for treatment control after focal tumor ablation, that (I) the operator can be fooled into believing that the achieved treatment zone is large enough to cover tumor and safety margin although in fact the treatment zone is too small (since Technique B overestimates the size of the treatment zone), and that (II) the correct positioning of the treatment zone in relation to the tumor can neither be confirmed nor denied objectively (since the barycenter offset is not quantified and visualized). Theoretically, the downloadable MITK freeware “Geometric evaluation of ablations“ allows everyone to determine whether the intended safety margin was realized, or if another focal tumor ablation is necessary to destroy completely the tumor. At this point, the authors are obliged to point out that the use of this software is currently limited to research, and clinical decisions based on this software prototype are currently strictly forbidden. In this study, we detected differences between Technique A and Technique B in regards of the geometry of the treatment zone. Clinical superiority for one technique, however, was not proven since no clinical data were analyzed.

This study has limitations. First, IRE was performed in normal pig liver tissue, and the extent of the treatment zone might be different in human tumor tissue (e.g. since different cell density). CT datasets from patients before and after IRE could be analyzed applying our approach, and the results correlated to local tumor control during follow-up. Second, only three different IRE protocols were used. Although those were clinically relevant, alternative protocols (e.g. different number and different spatial order of electrodes) might result in treatment zones more difficult to determine geometrically especially applying standard CT 3d analysis. IRE protocols are just now being optimized for different tumor sizes and tissue characteristics (e.g. liver cirrhosis), and then should undergo 3d analysis of the treatment zone. For such protocols, the benefits of CT 3d rendering with the “Chebyshev Center Concept” implemented could be even more obvious. Third, the presented results were collected in an acute setting. Survival studies should further evaluate subacute and chronic effects after IRE (e.g. imaging findings correlated to homogeneity of cell death). Since cellular repopulation and tissue healing is likely to occur after IRE of healthy liver tissue with complete resolution of the treatment zone over weeks, the clinical feasibility of our approach for acute treatment control could be best evaluated for liver tumors. Only if the tumor and safety margin is completely covered by the largest inscribed sphere within the treatment zone on acute post-interventional CT images and the tumor disappears during follow-up, our concept can be regarded as clinically relevant. Fourth, the comparison between Technique A and B is not on the same level since an automatic technique is compared with a manual technique. For a better comparison, an automation of Technique B would have been required.

## Conclusions

Regarding size and shape of the treatment zone after IRE, CT 3d rendering with the “Chebyshev Center Concept” implemented provides significantly different results compared with standard CT 3d analysis. Since standard CT 3d analysis cannot assess the extent of the treatment zone as precise as CT 3d rendering with the “Chebyshev Center Concept” implemented (especially since overestimation of the size of the treatment zone as well as the non-assessable position of the largest possible treatable tumor sphere within the treatment zone), the latter might be regarded as superior. The benefits could be used clinically to improve local tumor control due to a more objective acute treatment control.

## Competing interests

The authors declare that this study was supported technically and financially by AngioDynamics® Inc., Queensbury, USA.

## Authors’ contributions

CMS, BAR, SF and JW were responsible for the study concept. CMS, MFW, DV, SF, US, SZ and PLP participated in the concrete design of the study. CMS, DV, MBW, NB, SU, AS, DG, JK and SF carried out the experiments and/or performed the data acquisition. Quality control of data and algorithms was performed by MFW, MBW, JK and SZ. CMS, DV, MFW, SF, DG and AS participated in data analysis and interpretation. Statistical analysis was performed by CMS, NB, DV and MFW. The manuscript was prepared by CMS, DV and MFW. BAR, SU and SF were responsible for manuscript editing. PLP, HUK, BAR and JW reviewed the manuscript. All authors approved submission of the final manuscript.

## Pre-publication history

The pre-publication history for this paper can be accessed here:

http://www.biomedcentral.com/1471-2342/14/2/prepub
